# Infodemiology of West Nile Virus in Greece, 2024–2025, with Descriptive One Health Preparedness Evidence from Crete

**DOI:** 10.3390/epidemiologia7040102

**Published:** 2026-07-14

**Authors:** Antonios Papadakis, Eleftherios Koufakis, Sandra Gewehr, Spiros Mourelatos, Elias Ath Chaidoutis, George Pitsoulis, Apostolos Kamekis, Areti Lagiou

**Affiliations:** 1 Department of Public and Community Health, University of West Attica, 12243 Athens, Greece; 2 General Directorate of Public Health and Social Welfare of the Region of Crete, 71201 Heraklion, Greece; gpitsoulis@crete.gov.gr (G.P.); kamekis@yahoo.gr (A.K.); 3 Civil Protection Authority of the Region of Crete, 71201 Heraklion, Greece; 4 Ecodevelopment S.A., 57010 Thessaloniki, Greece; gewehr@ecodev.gr (S.G.);; 5 First Department of Pathology, School of Medicine, National and Kapodistrian University of Athens, 11527 Athens, Greece; echaidoutis@med.uoa.gr

**Keywords:** infodemiology, infoveillance, Google Trends, Wikipedia pageviews, West Nile virus, public health surveillance

## Abstract

Background/Objectives: West Nile virus (WNV) is a persistent mosquito-borne threat in Greece. This study examined whether online information-seeking patterns reflected official WNV surveillance during 2024–2025, with Crete providing descriptive field-level One Health preparedness context. Methods: Official national surveillance data were compared with country-level Google Trends relative search volume and Greek-language Wikipedia pageviews, focusing on weekly locally acquired West Nile neuroinvasive disease/central nervous system (WNND/CNS) cases. The Crete component separately summarized regional One Health preparedness. Results: Greece reported 220 locally acquired WNV cases (157 WNND/CNS) in 2024 and 96 (76 WNND/CNS) in 2025. Wikipedia pageviews showed the strongest full-year associations with WNND/CNS activity when pageviews followed cases by one week (2024: Spearman rho = 0.802; 2025: rho = 0.763; both *p* < 0.001). Google Trends showed weaker associations at the same lag (2024: rho = 0.402, *p* = 0.003; 2025: rho = 0.452, *p* < 0.001). Transmission-period sensitivity analyses attenuated several associations: the 2025 Wikipedia associations and the Google Trends associations in both years were not statistically significant. The first-difference lag analysis identified no leading digital signal. Conclusions: Wikipedia showed more stable language-specific temporal concordance with national surveillance than Google Trends. However, the digital indicators reflected concurrent or lagging public attention and did not demonstrate predictive capacity. The Crete component separately illustrates how regional One Health preparedness complements national surveillance and risk communication.

## 1. Introduction

West Nile virus (WNV) remains a primary mosquito-borne viral threat in Europe. During the 2024 transmission season, 19 European countries reported 1436 locally acquired human cases; by early December 2025, 1112 locally acquired human cases and 97 deaths had been reported. These figures highlight the persistent public health relevance of WNV and the need for surveillance strategies that integrate epidemiological data, vector monitoring, and timely analysis of public attention [1].

In the Mediterranean basin, mosquito-borne disease risk is increasing. Climate change, land-use change, globalization, and ecological disruption are increasingly recognized as major drivers of mosquito-borne disease risk, influencing vector distribution, seasonal activity, host–vector contact, and the probability of local transmission. In Mediterranean and European settings, warmer conditions may extend vector activity periods, support prolonged mosquito survival and continued breeding activity, and facilitate the establishment or expansion of invasive *Aedes* species [2,3,4,5,6,7,8,9,10,11]. Additionally, the presence of invasive species such as *Aedes aegypti* and *Aedes albopictus* raises the stakes for potential outbreaks of dengue, chikungunya, and Zika [12,13,14,15,16,17,18].

In addition, mosquito-borne disease risk is shaped not only by vector ecology and climate, but also by travel, tourism, migration, cross-border mobility, and arrivals from endemic settings. Greece is a relevant setting for examining these issues because official human surveillance data are available nationally and because preparedness extends beyond a single pathogen. Because WNV recurs seasonally in Greece, it requires coordinated mosquito control, clinical vigilance, risk communication, and blood safety measures. At the same time, Greece’s successful elimination of endemic malaria represents an important public health achievement and a valuable international example, while continued vigilance remains necessary for imported cases and sporadic events with evidence of local transmission in the post-elimination period [19]. Imported dengue, chikungunya, and Zika virus disease events also remain relevant for travel medicine, clinical awareness, and preparedness, even if they do not generate domestic transmission or comparable domestic temporal series [20,21,22,23,24,25,26,27,28].

The long-term national burden further supports the public health relevance of WNV in Greece. According to aggregated national surveillance data from the Hellenic National Public Health Organization (NPHO), 2184 WNV cases were reported in Greece during 2010–2025, including 1547 cases with central nervous system involvement and 301 deaths. This historical pattern highlights the recurrent severity of WNV in Greece and supports the use of WNND/CNS cases as a stable clinical anchor for the present infodemiological analysis. Among these CNS cases, encephalitis or meningoencephalitis predominated (89%), the burden was concentrated in older adults (≈82% aged ≥60 years), and case fatality among CNS cases reached approximately 19%, underscoring the clinical severity and high surveillance visibility that make WNND/CNS a suitable epidemiological anchor for the present analysis [29].

The digital transformation of society has changed the public health information environment. While traditional surveillance remains essential, digital traces from online search activity and pageviews can provide complementary insight into population-level attention, perceived risk, and public response during health events [30,31,32,33,34]. From a public health perspective, identifying periods of intensified online attention can help authorities time educational messages, address misconceptions, and align risk communication with moments when the public is actively seeking information, even when these signals are not predictive of future case occurrence. The COVID-19 pandemic demonstrated that monitoring disease occurrence alone is not sufficient for effective public health communication. Therefore, infoveillance must account for public information-seeking behaviors as well as the spread of misinformation, disinformation, and malinformation. In the context of mosquito-borne diseases, infodemiology can help identify periods of intensified public attention, provided that digital indicators are interpreted alongside official epidemiological data and within the specific public health context of each pathogen [35,36,37,38,39,40].

Google Trends and Wikipedia pageviews are common indicators in this field, yet they capture different behaviors. Google Trends provides relative search volume, while Wikipedia pageviews often reflect more sustained information-seeking. In the Greek setting, WNV provides the clearest context for inferential infodemiological analysis because it combines recurrent seasonal occurrence, official national surveillance data, and direct operational implications for public health [41,42,43,44,45,46,47,48,49,50].

The local preparedness component of the present study was included to provide field-level context for WNV preparedness and response. Crete represents a Mediterranean island setting of public health relevance, because it combines climate-sensitive mosquito ecology, intense tourism, seasonal population turnover, points of entry, arrivals from endemic regions, and structured human, entomological, laboratory, and animal-sentinel surveillance. In this two-level structure, country-level Google Trends and Greek-language Wikipedia data were utilized to examine online information-seeking patterns, whereas the regional framework in Crete was used to illustrate field-level preparedness capacity.

Against this background, the present study examined whether digital information-seeking activity reflected official WNV surveillance patterns in Greece during 2024–2025. The inferential analysis focused on weekly locally acquired West Nile neuroinvasive disease/central nervous system (WNND/CNS) cases, while malaria, dengue, chikungunya, and Zika virus disease were retained only as descriptive epidemiological context. The specific aims were: (i) to describe official human mosquito-borne infection occurrence in Greece, (ii) to analyze temporal patterns in Google Trends and Greek-language Wikipedia pageviews for WNV, (iii) to examine associations between digital attention indicators and official weekly WNND/CNS surveillance data, and (iv) to contextualize the national findings using local preparedness evidence from Crete, including human surveillance events, entomological monitoring, molecular testing, sentinel chicken serosurveillance, winter mosquito activity, operational response data, national risk-classification context, and citizen-science contextual information.

To our knowledge, few studies in Greece have examined national infodemiological signals alongside field-level One Health preparedness data for WNV [36,37,51,52,53,54,55,56,57]. In the present study, digital attention indicators are interpreted together with descriptive regional preparedness evidence from Crete, offering a two-level perspective on surveillance, risk communication, and operational readiness.

## 2. Materials and Methods

### 2.1. Study Design

This infodemiology study examined whether digital information-seeking activity reflected official national surveillance patterns for WNV in Greece during 2024–2025. The inferential analysis focused on WNV, specifically on locally acquired WNND/CNS cases.

Google Trends and Greek-language Wikipedia pageviews were used as complementary digital attention indicators. While commercial social listening platforms (e.g., Talkwalker, Brandwatch) can capture general online discourse, Google Trends and Wikipedia were explicitly selected for this study because they reflect active, deliberate information-seeking behavior rather than passive social media sharing. Furthermore, they provide open-access data that support methodological transparency and have been widely used within established infoveillance frameworks [37,38]. Because Google Trends data were extracted at the country level and Greek-language Wikipedia pageviews cannot be reliably localized, the digital indicators were compared only with the national surveillance series, while Wikipedia pageviews were interpreted as a language-specific rather than geographically restricted measure. Weekly digital indicators were aligned with official WNND/CNS surveillance data to examine temporal concordance between online information-seeking activity and severe WNV case occurrence.

A descriptive local preparedness component from the Region of Crete was included to illustrate how digital attention indicators evaluated against national surveillance can be interpreted alongside a field-level operational preparedness model. This component integrated aggregated human surveillance events, routine and winter entomological monitoring, molecular testing of mosquitoes, sentinel chicken serosurveillance, mosquito-control indicators, operational response metrics, and citizen-science contextual information. The Crete component is presented as descriptive field-level context and was not statistically integrated with the national digital indicators.

### 2.2. Human Epidemiological Data

Official human surveillance data were obtained from publicly available surveillance reports and epidemiological documents issued by the NPHO. For both the 2024 and 2025 transmission seasons, the inferential analysis utilized the weekly distribution of locally acquired West Nile neuroinvasive disease/central nervous system (WNND/CNS) cases by symptom onset as the epidemiological reference series.

This clinical category was selected to improve methodological consistency between 2024 and 2025 and to focus the analysis on severe WNV events that are systematically reported and likely to have greater public health visibility. All imported WNV cases, as well as non-CNS cases, were explicitly excluded from the inferential digital analysis. Regarding other mosquito-borne infections, official national occurrence data for malaria, dengue, chikungunya, and Zika virus disease were retrieved solely to provide a descriptive epidemiological context for the study period, without being subjected to temporal statistical analysis.

### 2.3. Digital Search Data

Digital information-seeking data were obtained from two sources: Google Trends [58] and the Wikimedia representational state transfer (REST) Pageviews application programming interface (API) [59], which was used to retrieve Greek-language Wikipedia pageviews. Data covered the period from 1 January 2024 to 31 December 2025. Both sources were used as complementary infodemiological indicators to examine temporal patterns of public online attention related primarily to WNV.

Google Trends data were extracted at the country level for Greece (geo = GR) using the Web Search property and the search-term option (not the topic/entity option), with the category filter set to all categories (code 0).

The country level was selected a priori to match the geographic resolution of the official weekly WNND/CNS surveillance series used as the epidemiological comparator. Although Google Trends may display subnational interest in some settings, the availability and stability of regional estimates depend on query volume and platform reporting thresholds; regional data were therefore not used for inference in this study. Google Trends provides normalized relative search volume (RSV) values ranging from 0 to 100, where a value of 100 represents the peak popularity of a search term within the selected time frame and geographic setting. An RSV value of 0 indicates insufficient search volume for the term to be assigned a value above zero within the Google Trends normalization procedure and does not necessarily represent a complete absence of searches. Weekly values were analyzed as returned, without imputation or smoothing. Weekly RSV time series were extracted for the Greek WNV-related terms “Ιός Δυτικού Νείλου” and “ιός του Δυτικού Νείλου”, as well as for the English term “West Nile virus”, separately for the periods 1 January–31 December 2024 and 1 January–31 December 2025. The Greek terms “Ιός Δυτικού Νείλου” and “ιός του Δυτικού Νείλου” both translate as “West Nile virus”; the former reflects a shorter, commonly used search expression, whereas the latter corresponds more closely to the formal Greek article title used by Wikipedia. Rolling averages were not applied because the weekly resolution matched the epidemiological surveillance series, and smoothing the highly intermittent Google Trends data could obscure short-lived peaks without resolving the underlying low-volume limitation. These terms were selected a priori because they represent the most direct Greek-language expressions for WNV and allowed comparison between common search behavior and more formal information-seeking. The English term “West Nile virus” was also retained to capture possible English-language search behavior within Greece. Regional linguistic variants were not included in the a priori query set. Socioeconomic status and literacy could not be assessed because Google Trends provides aggregate, anonymized data without user-level demographic information. The analytical Google Trends series used in this study were retrieved on 6 May 2026, using a separate retrieval for each term and study year. Because Google Trends returns sampled relative search volume series that may vary between retrievals, reliance on a single retained series per term and year is acknowledged as a reproducibility limitation.

Greek-language Wikipedia pageview data were retrieved through the Wikimedia REST Pageviews API for the article “Ιός του Δυτικού Νείλου” on the Greek Wikipedia project (https://el.wikipedia.org, accessed on 6 February 2026), using the parameters access = all-access (including desktop, mobile web, and mobile applications), agent = user (excluding automated agents and crawlers), and granularity = daily. Daily counts were subsequently aggregated into weekly totals to match the temporal resolution of the surveillance and Google Trends series. Because the data were retrieved retrospectively after completion of both study periods, API processing or retrieval latency did not alter the calendar date assigned to each daily pageview count and was not modeled as an additional lag. The weekly Wikipedia series was used as a complementary indicator of more deliberate or sustained information-seeking activity. Because reliable per-article reader geolocation is not publicly available for Wikimedia pageviews, the Greek-language Wikipedia series could not be geographically restricted to Greece. It was therefore interpreted as a language-specific indicator of information-seeking activity rather than as a strictly Greece-localized measure of online attention.

Additional Google Trends and Wikipedia data for malaria, dengue, chikungunya, and Zika virus disease were collected only for descriptive context. Thus, the inferential digital–epidemiological analysis was restricted to WNV.

### 2.4. Local Surveillance and Preparedness Data from Crete

Descriptive local surveillance and preparedness data from Crete were supplied by the investigators and were used to provide field-level operational context for infodemiological analysis based on national surveillance data. All field-level entomological and operational data used in this study were derived from field data collected during 2024 and 2025 by the comprehensive mosquito-control and entomological surveillance programs officially implemented by the Public Health Authority of the Region of Crete. This local component included aggregated human mosquito-borne infection events, routine and winter entomological surveillance records, mosquito-control operational indicators, molecular testing results from selected mosquito samples, sentinel chicken serosurveillance, operational response information, and citizen-science contextual information.

Operational mosquito-control indicators were derived from field data collected during 2024 and 2025. These included the number of major wetland systems monitored, wetland sampling checks, wetland spraying interventions, settlements covered, peri-urban and rural inspection visits, breeding sites inspected, peri-urban and rural spraying interventions, stormwater drains inspected, large-city operational rounds, and citizen requests addressed. These indicators were used to describe the scale and continuity of the local mosquito-control and preparedness program.

Although the primary epidemiological focus of this study is WNV, a robust regional preparedness program must address the broader spectrum of mosquito-borne threats. Therefore, an important component of the surveillance framework in Crete is the systematic monitoring of potential points of entry, specifically international airports and major seaports that serve as points of entry. This targeted strategy focuses on the early detection of invasive mosquito species, primarily *Aedes aegypti*, following national health directives that identify these locations as high-risk gateways for vector introduction. Furthermore, in accordance with the legislative framework established by Joint Ministerial Decision 23247/2025 (Government Gazette 3041/B/19 June 2025), the program incorporates the verification of disinsection certificates for aircraft arriving from countries or regions with established *Aedes aegypti* populations or other specified risk areas [60]. These mandatory measures apply to civil and military aircraft, subject to the exceptions specified in the Decision, and aim to prevent the accidental importation and subsequent establishment of competent vectors for dengue, Zika virus disease, and chikungunya.

The routine entomological dataset covered 2024 and 2025 and comprised adult mosquito sampling events conducted twice monthly from March to November each year at fixed sampling sites across Crete. Sampling was performed using custom-built traps constructed and operated by Ecodevelopment S.A. (Thessaloniki, Greece), the contractor responsible for the regional mosquito-control program. The trap types used in the program were identified by the contractor as “Ecodev traps” and “Triple Traps.” The Ecodev traps used CO_2_ as an attractant, supplied from CO_2_ bottles at a constant flow of 500 mL/min, to attract host-seeking adult female mosquitoes, whereas the Triple Traps functioned as photocatalytic UV-light traps. Specimen counting and morphological identification at species level were performed by Ecodevelopment S.A. using dichotomous taxonomic keys for European and Greek mosquitoes [61,62]. The routine entomological surveillance records provided species-level identification results.

Operational preparedness in Crete was further supported by the Early Warning System for Mosquito-borne Diseases (EYWA, v2.7.1), developed and coordinated by the BEYOND Centre of Earth Observation Research and Satellite Remote Sensing of the National Observatory of Athens. Rather than performing primary modeling, the regional authorities utilized the operational outputs provided by the EYWA platform. Specifically, the regional framework integrated the risk classifications generated by MAMOTH, an Earth observational data-driven model for mosquito abundance prediction, and MIMESIS, a spatial dynamic model for WNV. The detailed predictive architecture, Earth observation integration, and validation of these models have been previously described by their developers [63,64,65,66,67,68,69,70]. Complementing these epidemiological early warnings, the Mosquito Vision smartphone application delivers settlement-level mosquito nuisance predictions to the public, based on Big Data Technologies’ model for Adult mosquitoes (Bad, v2.7.1) developed by Ecodevelopment (also an EYWA product), serving as a supplementary tool for the region’s proactive risk management [71].

An additional winter entomological surveillance dataset covered the period from January to March 2024. This survey used fixed mosquito traps, with emphasis on urban areas, ports, airports, and other points of entry. Sampling was conducted every 15 days. Species identification was performed at the species level, and meteorological variables were recorded during trap deployment.

Selected mosquito samples from the local surveillance program underwent molecular testing at the Institute of Molecular Biology and Biotechnology of the Foundation for Research and Technology Hellas (IMBB-FORTH) for WNV, Usutu virus, chikungunya virus, and Zika virus. These molecular findings served as selected arbovirus testing to support the broader local preparedness program. IMBB-FORTH also performed genus- and species-level identification of the examined mosquitoes.

Sentinel chicken serosurveillance was included as an animal-sentinel component of local WNV preparedness and was interpreted as an environmental indicator of possible WNV circulation in the local transmission cycle. The surveillance system was based on serum samples collected from free-range sentinel chickens in the Region of Crete. Samples were tested using a competitive enzyme-linked immunosorbent assay (ELISA) for the detection of WNV-specific antibodies ID Screen West Nile Competition (IDVet, Grabels, France). Results were interpreted operationally according to a predefined risk-classification scheme based on the number and distribution of WNV-positive chicken samples. The risk level was classified as zero when no positive samples were detected, low when one positive sample was detected in one poultry holding within a broader control area, moderate when one positive sample per poultry holding was detected in multiple holdings within a broader control area, high when two to three positive samples per poultry holding were detected in multiple holdings, and very high when more than three positive samples per poultry holding were detected in multiple holdings. These findings were used descriptively as field-level animal-sentinel evidence within the regional preparedness framework. Assay positivity was determined according to the manufacturer’s instructions. The available dataset comprised aggregated operational results from two sampling cycles per year. The number and coordinates of individual holdings or sampling sites, exact sampling dates, repeated-bird identifiers, exact ages beyond classification as young chickens, and individual chicken-level serological results were not available for analysis. Accordingly, the sentinel findings were interpreted descriptively and were not used for individual-level, spatial, or temporal inference.

Citizen-science contextual information was derived from the Mosquito Vision^®^ application (v2.7.1), which allowed citizens to report potential mosquito breeding sites and nuisance levels and to receive information on expected mosquito nuisance. These data were used as part of the local preparedness context and were not analyzed as a standalone citizen-science dataset.

### 2.5. Local Response to a Human WNV Case in Crete

An anonymized local WNV operational response component was included to describe how regional preparedness procedures were activated following notification of a locally acquired human WNV case in Crete during 2025. The information was derived from operational public health response records and was fully anonymized. No patient-level clinical information, demographic characteristics, exact residence details, settlement names, or other potentially identifying information were included.

This notification was operationally important because it represented the first locally acquired human WNV event recorded in Crete since 2018. Under the national WNV action framework of the Ministry of Health, notification of a locally acquired human WNV case triggers risk-based classification and response procedures for the relevant municipality, including measures related to affected or high-risk areas and blood safety.

The response was interpreted in relation to the national WNV action framework, which links human surveillance, animal and bird surveillance, entomological surveillance, mosquito-control measures, blood safety measures, intersectoral communication, risk assessment, and graded prevention and response activities.

In this study, the operational response component was used to illustrate the practical translation of preparedness procedures into field action and was not treated as a clinical case report or an epidemiological case series.

### 2.6. Statistical Analysis

The statistical analysis examined temporal associations between official weekly WNV surveillance data in Greece and two digital attention indicators: Google Trends RSV and Greek-language Wikipedia pageviews. Inferential analysis focused on locally acquired WNND/CNS cases, because this case category provided a consistent epidemiological reference series across 2024 and 2025 and represented clinically severe WNV activity with high public health visibility.

Weekly WNND/CNS case counts were compared with weekly Google Trends RSV values and weekly Greek-language Wikipedia pageviews for the same study period. Google Trends analyses included the Greek WNV-related terms “Ιός Δυτικού Νείλου” and “ιός του Δυτικού Νείλου”, as well as the English term “West Nile virus”. Greek-language Wikipedia pageviews for “Ιός του Δυτικού Νείλου” were aggregated from daily counts into weekly totals to match the temporal resolution of the epidemiological data.

Because epidemiological and digital variables were expected to show seasonal concentration, non-normal distributions, and multiple zero values, Spearman’s rank correlation coefficient (rho) was selected as the primary measure of association. Pearson’s correlation coefficient (r) was also calculated as a secondary parametric measure to describe linear associations. Correlation coefficients were interpreted descriptively as weak (<0.30), moderate (0.30–0.69), or strong (≥0.70). Two-sided nominal *p*-values were reported. To account for multiple comparisons and control the proportion of Type I errors, the Benjamini–Hochberg False Discovery Rate (FDR) procedure was applied to all correlation analyses, with statistical significance assessed at an FDR-adjusted threshold of 0.05.

The main pairwise analyses included comparisons between weekly WNND/CNS cases and Google Trends RSV, weekly WNND/CNS cases and Greek-language Wikipedia pageviews, and Google Trends RSV and Wikipedia pageviews. Lag analyses were additionally performed to explore temporal relationships between epidemiological activity and digital attention. Associations were examined for the same week (lag 0), for digital indicators preceding reported WNND/CNS cases by one week, and for digital indicators following reported WNND/CNS cases by one week. The ±1-week lag window was selected a priori because the analysis used weekly epidemiological surveillance data and aimed to explore short-term temporal alignment between public information-seeking and reported WNND/CNS activity, rather than to build a forecasting model. Longer distributed-lag structures were not examined because the study period included only two annual transmission cycles and sparse weekly case counts, which limited the stability of more complex lag models.

Analyses were conducted separately for 2024 and 2025. The two annual series were not pooled because the magnitude and seasonal distribution of WNV activity differed between the two transmission seasons.

To assess whether full-year correlations were influenced by shared seasonality and weeks with zero or minimal activity, we performed an additional sensitivity analysis restricted to the observed WNV transmission period for each year. This period was defined from the first to the last epidemiological week with reported locally acquired WNV activity: weeks 25–44 for 2024 and weeks 26–40 for 2025. The sensitivity analysis evaluated whether the direction and relative pattern of associations between WNND/CNS cases and digital indicators remained consistent after excluding non-epidemic weeks. As a further exploratory robustness check of the short transmission-period series, each series was first-differenced to reduce the influence of shared trends and short-term autocorrelation, and lagged Spearman rank correlations between the differenced series were examined at lags from −2 to +2 weeks. Given the limited number of weekly observations, the results were interpreted descriptively and were not used for formal statistical inference. This first-difference lag analysis was not considered formal autoregressive integrated moving average (ARIMA) pre-whitening or a forecasting analysis.

Non-CNS WNV cases were described epidemiologically but were not included in the primary inferential analysis. Local preparedness data from Crete, including operational response information, sentinel chicken findings, mosquito-control indicators, molecular testing results, and citizen-science contextual information, were summarized descriptively as field-level preparedness evidence. All analyses were conducted using Epi Info v7.2.7.0 (Centers for Disease Control and Prevention, Atlanta, GA, USA).

## 3. Results

### 3.1. Official Human West Nile Virus Surveillance Data in Greece, 2024–2025

Official national surveillance data showed that WNV provided the most suitable epidemiological series for temporal analysis in Greece during the study period. In 2024, Greece recorded 226 human WNV cases, of which 220 were locally acquired and 6 were imported. Among the locally acquired cases, 157 presented with central nervous system manifestations and 63 did not present with central nervous system manifestations. Thirty-five deaths were reported among hospitalized patients with WNV infection. The first symptomatic case of the 2024 transmission season had symptom onset in epidemiological week 25, and the last diagnosed case was reported in epidemiological week 44, indicating seasonal activity from June to October.

In 2025, official WNV surveillance documented continued seasonal activity in Greece. Up to the final weekly surveillance report of 3 December 2025, 96 locally acquired human WNV cases had been diagnosed and investigated, including 76 cases with central nervous system manifestations and 20 cases without central nervous system manifestations. Two additional imported WNV cases were reported and were excluded from the domestic-case analysis. Nine deaths were recorded among patients with WNV infection and central nervous system manifestations. The first recorded 2025 case had symptom onset in epidemiological week 26, and the last diagnosed case had symptom onset in epidemiological week 40, indicating seasonal activity from late June to late September.

For descriptive epidemiological context, malaria was reported mainly as an imported disease in both study years, and four imported dengue cases were reported in 2024.

Table 1 summarizes the official national human WNV surveillance data for 2024–2025. Overall, WNV showed the clearest seasonal and temporal structure, and the inferential analysis therefore focused on WNV, specifically on WNND/CNS cases.

### 3.2. Infodemiology Analysis for West Nile Virus, 2024–2025

For both 2024 and 2025, the infodemiology analysis used weekly locally acquired WNND/CNS cases as the epidemiological reference series. This approach ensured methodological consistency between the two study years. Weekly Google Trends RSV values and weekly Greek-language Wikipedia pageviews were aligned with the official weekly WNND/CNS surveillance data.

In 2024, the weekly WNND/CNS case series included 157 locally acquired cases. Weekly case activity was positively associated with Greek-language digital attention indicators. For the Google Trends term “Ιός Δυτικού Νείλου”, the same-week association with WNND/CNS cases was statistically significant but moderate (Spearman rho = 0.351, *p* = 0.010; Pearson r = 0.313, *p* = 0.022). The association remained similar when digital activity preceded cases by one week (Spearman rho = 0.348, *p* = 0.011) and was slightly stronger when digital activity followed cases by one week (Spearman rho = 0.402, *p* = 0.003). For the Google Trends term “ιός του Δυτικού Νείλου”, the same-week Spearman association was weaker and did not reach statistical significance (Spearman rho = 0.252, *p* = 0.069), although Pearson correlation suggested a statistically significant linear association (Pearson r = 0.318, *p* = 0.020). The English Google Trends term “West Nile virus” could not be evaluated for 2024 because the extracted weekly RSV series contained only zero values.

Greek-language Wikipedia pageviews for “Ιός του Δυτικού Νείλου” showed stronger and more consistent associations with weekly WNND/CNS activity in 2024. The same-week association was strong and statistically significant (Spearman rho = 0.750, *p* < 0.001; Pearson r = 0.816, *p* < 0.001). Lag analysis showed that the association remained strong when Wikipedia activity preceded cases by one week (Spearman rho = 0.714, *p* < 0.001) and was strongest when Wikipedia pageviews followed WNND/CNS cases by one week (Spearman rho = 0.802, *p* < 0.001).

In 2025, the weekly WNND/CNS case series included 76 locally acquired cases. The Google Trends term “Ιός Δυτικού Νείλου” again showed a statistically significant moderate association with weekly WNND/CNS activity at lag 0 (Spearman rho = 0.418, *p* = 0.002; Pearson r = 0.396, *p* = 0.003). The association was similar when digital activity preceded cases by one week (Spearman rho = 0.410, *p* = 0.003) and slightly stronger when digital activity followed cases by one week (Spearman rho = 0.452, *p* < 0.001). For the Google Trends term “ιός του Δυτικού Νείλου”, the same-week association was weak and not statistically significant by Spearman correlation (Spearman rho = 0.245, *p* = 0.077), although Pearson correlation showed a nominal association (Pearson r = 0.271, *p* = 0.049) that did not remain significant after FDR correction. A statistically significant Spearman association was observed when digital activity followed WNND/CNS cases by one week (Spearman rho = 0.297, *p* = 0.032). The English Google Trends term “West Nile virus” did not show a meaningful association with WNND/CNS activity in 2025.

Wikipedia pageviews again provided the most consistent digital attention signal in 2025. Weekly Greek-language Wikipedia pageviews for “Ιός του Δυτικού Νείλου” were strongly associated with weekly WNND/CNS cases at lag 0 (Spearman rho = 0.718, *p* < 0.001; Pearson r = 0.630, *p* < 0.001). The association remained strong when Wikipedia activity preceded cases by one week (Spearman rho = 0.701, *p* < 0.001) and was strongest when Wikipedia pageviews followed WNND/CNS cases by one week (Spearman rho = 0.763, *p* < 0.001).

Overall, the full-year weekly analysis showed that Greek-language Wikipedia pageviews were the most stable digital indicator of WNND/CNS activity across both years. Google Trends showed weaker but statistically significant associations for the Greek search term “Ιός Δυτικού Νείλου”, whereas the more formal Greek phrase “ιός του Δυτικού Νείλου” showed less consistent performance. The strongest associations for Wikipedia were observed when pageviews followed WNND/CNS cases by one week. This temporal pattern suggests that Wikipedia activity was more likely to reflect sustained or explanatory information-seeking after periods of increased epidemiological visibility, rather than an early predictive signal. Table 2 summarizes the weekly correlation results.

**Figure 1:**
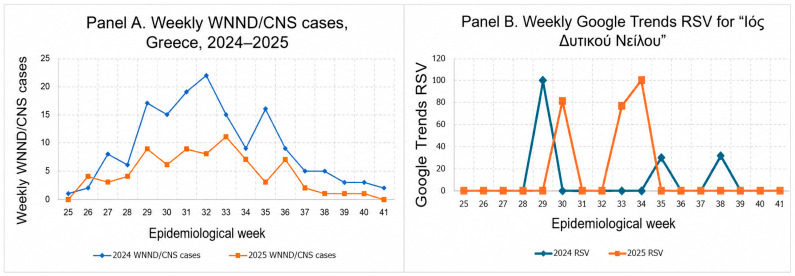
Weekly locally acquired WNND/CNS cases and Google Trends relative search volume (RSV) for “Ιός Δυτικού Νείλου” in Greece, 2024–2025. Panel (**A**) presents the weekly WNND/CNS case series for 2024 and 2025, and Panel (**B**) presents the corresponding Google Trends RSV series.

In the sensitivity analysis restricted to the observed WNV transmission period, the magnitude of several associations was attenuated compared with the full-year analysis. For Wikipedia pageviews, the association with WNND/CNS cases remained evident in 2024 at lag 0 (Spearman rho = 0.675; *p* = 0.001) and was strongest when pageviews followed cases by one week (Spearman rho = 0.843; *p* < 0.001). In 2025, however, the corresponding transmission-period associations were substantially weaker and did not reach statistical significance (lag 0: Spearman rho = 0.036; *p* = 0.899; pageviews following cases by one week: Spearman rho = 0.441; *p* = 0.100). Google Trends associations were also attenuated in the transmission-period analysis and were not statistically significant in either year. These findings suggest that shared seasonality and non-epidemic weeks contributed to the magnitude of some full-year correlations, supporting a more cautious interpretation of digital indicators as complementary measures of public information-seeking rather than predictive epidemiological tools. The exploratory first-difference lag analysis identified no leading digital signal: Wikipedia activity remained concurrent with or lagged behind reported cases, whereas Google Trends correlations were unstable and non-significant. Figure 1 presents weekly locally acquired WNND/CNS cases and Google Trends relative search volume for “Ιός Δυτικού Νείλου” during the 2024 and 2025 WNV transmission seasons in Greece. The figure illustrates the seasonal concentration of neuroinvasive WNV cases and the more intermittent pattern of Google search activity.

**Figure 2:**
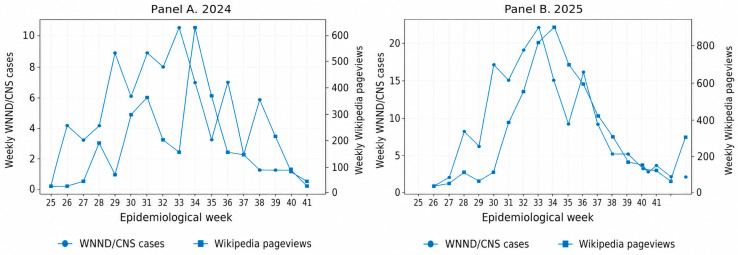
Weekly locally acquired WNND/CNS cases and Greek-language Wikipedia pageviews for “Ιός του Δυτικού Νείλου” in Greece, 2024–2025. Panel (**A**) shows the 2024 weekly series, and Panel (**B**) shows the 2025 weekly series. Wikipedia pageviews were aggregated from daily counts into weekly totals.

Figure 2 presents weekly locally acquired WNND/CNS cases and Greek-language Wikipedia pageviews for “Ιός του Δυτικού Νείλου” during the same period. Temporal correspondence was evident in 2024 but less consistent in 2025, in agreement with the transmission-period sensitivity analysis.

### 3.3. Local Preparedness Evidence from Crete, 2024–2025

Local preparedness evidence from Crete documented a comprehensive mosquito-control and surveillance structure during 2024–2025. The program covered the whole Region of Crete and included activities relevant to WNV preparedness and broader arboviral risk management, including larval surveillance of major wetland systems, peri-urban and rural breeding sites, urban stormwater drains, adult mosquito trapping, molecular testing of selected mosquito samples, sentinel chicken serosurveillance, response to citizen requests, and citizen-facing reporting through the Mosquito Vision^®^ application.

In 2024, the program included 9 major wetland systems, 747 settlements, more than 71,420 stormwater drains, and 576 adult mosquito samplings at 32 fixed trap sites. In 2025, it included 13 major wetland systems, 741 settlements, more than 70,284 stormwater drains, and 608 adult mosquito samplings at 32 fixed and 22 ad hoc trap sites. These indicators show the scale and continuity of the local mosquito-control and preparedness program across both years. No WNV-positive mosquito pools were detected in either year among the tested mosquito samples. Sentinel chicken serosurveillance was conducted as an animal-sentinel component of local WNV preparedness. In 2024, 504 samples from young domestic chickens were tested across two sampling cycles, and no WNV-seropositive sentinel chickens were detected. In 2025, 511 samples were tested across two sampling cycles; no WNV-seropositive sentinel chickens were detected in the first cycle, whereas eight WNV-seropositive sentinel chickens were detected in the second cycle. These findings indicated localized animal-sentinel evidence of environmental WNV activity in 2025. Local human surveillance events in Crete included one imported malaria case in 2024, three imported malaria cases in 2025, one imported dengue case in 2025, and one locally acquired WNV case in 2025. These events were used as operational preparedness indicators within the regional surveillance framework. Citizen-science contextual information was available through the Mosquito Vision^®^ application, which allowed citizens to report potential mosquito breeding sites and nuisance levels and to receive information on expected mosquito nuisance.

During the study period, the local operational response was guided by the risk assessments provided by the EYWA system. According to the system’s operational outputs, the MAMOTH model indicated a relatively stable pattern for October 2024, with mosquito populations predominantly classified within low and moderate risk categories across the monitored area. In contrast, the system’s updates for October 2025 identified a marked shift, mapping specific, localized grid cells in the high-risk category for *Culex* spp. populations. Simultaneously, the MIMESIS model outputs for late 2025 indicated an elevated localized probability of WNV occurrence. These outputs were used operationally to support preparedness awareness and targeted field vigilance. No prospectively defined prediction time, spatial-grid match, probability threshold, or anonymized case-location validation was performed; therefore, the outputs were not interpreted as validated predictions of human WNV occurrence. These operational actions are further summarized in Table 3.

### 3.4. Local Vector Activity Indicators from Crete

Local vector activity indicators from Crete complemented the preparedness evidence described above and provided field-level context for interpreting WNV risk. Larval surveillance showed that positivity patterns differed by mosquito genus during 2025 compared with the historical reference period. For *Culex* spp., larval positivity remained generally lower than the 2021–2024 average during most surveillance weeks. For *Aedes* spp. and *Anopheles* spp., larval positivity was also lower than the corresponding historical averages for much of the season. These findings were consistent with the operational assessment that mosquito nuisance remained at low levels during the implementation period.

Adult mosquito surveillance showed a similar pattern. Female *Culex* spp. abundance in 2025 was generally comparable to the 2020–2024 average. Female *Aedes* spp. abundance was lower than the historical average during much of the season, although a late-summer increase was observed and reached levels comparable to, or locally exceeding, the historical reference pattern. Female *Anopheles* spp. abundance was generally comparable to previous years. Overall adult mosquito abundance in 2025 was slightly lower than the 2020–2024 average. These indicators are important because low or average vector abundance does not exclude focal WNV circulation or localized transmission risk. In Crete, the combination of larval surveillance, adult mosquito monitoring, molecular testing, sentinel chicken serosurveillance, and targeted field response provided a broader preparedness framework than any single indicator alone. This is particularly relevant in island settings with intense seasonal mobility, points of entry, and heterogeneous local ecological conditions.

**Figure 3:**
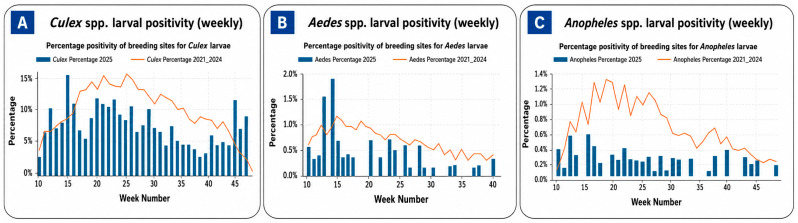
Weekly larval positivity indicators for *Culex* spp., *Aedes* spp., and *Anopheles* spp. in Crete during 2025 compared with the 2021–2024 average. Panels (**A**–**C**) show weekly larval positivity patterns for *Culex* spp., *Aedes* spp., and *Anopheles* spp., respectively.

Figure 3 summarizes the 2025 larval positivity patterns in Crete relative to the 2021–2024 average.

**Figure 4:**
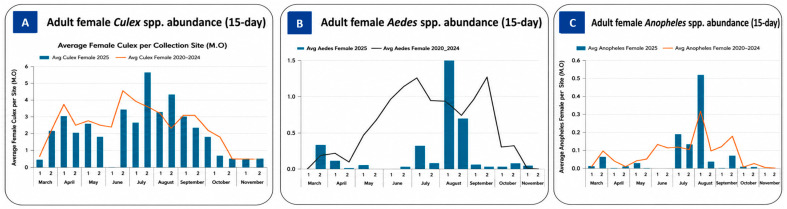
Adult female mosquito abundance indicators for *Culex* spp., *Aedes* spp., and *Anopheles* spp. in Crete during 2025 compared with the 2020–2024 average. Panels (**A**–**C**) show *Culex* spp., *Aedes* spp., and *Anopheles* spp., respectively.

Figure 4 summarizes adult female abundance indicators for *Culex* spp., *Aedes* spp., and *Anopheles* spp. in Crete during 2025 relative to the 2020–2024 average. These data are included as descriptive field-level preparedness evidence within the regional surveillance framework, although the current domestic WNV threat is primarily associated with *Culex* spp.

## 4. Discussion

This study examined whether digital information-seeking activity reflected official West Nile virus (WNV) surveillance in Greece during 2024–2025, with the inferential analysis focused on weekly locally acquired WNND/CNS cases. This focus is also supported by long-term national surveillance data, which show that WNV cases with central nervous system involvement constitute a substantial proportion of reported WNV disease in Greece and represent the clinically severe component most consistently captured by surveillance systems. The main finding was that WNV showed the clearest and most epidemiologically interpretable digital signal in this dataset. In the full-year weekly analysis, WNND/CNS activity was consistently associated with Greek-language Wikipedia pageviews in both years, whereas Google Trends showed weaker but statistically significant associations for the Greek search term “Ιός Δυτικού Νείλου”. This pattern is consistent with the recurrent seasonality of WNV, its operational relevance for mosquito control and blood safety, and its public health visibility during the transmission season.

Previous literature supports this pathogen-specific interpretation. Provenzano et al. showed that Wikipedia-based information-seeking activity correlated with conventional arboviral surveillance data in Italy, supporting the use of pageviews as complementary indicators of public attention [72]. Santangelo et al. reported similar associations between official surveillance data and Google Trends or Wikipedia indicators for major arboviral diseases in Italy, while also showing that the strength of these associations varied by pathogen and platform [73]. More broadly, Mavragani and Ochoa emphasized the need for careful search-term selection, geographic specification, language awareness, and cautious interpretation of normalized Google Trends data [38]. Carneiro and Mylonakis also highlighted the value of Google Trends for timely disease surveillance, while stressing that search activity should be interpreted alongside conventional surveillance rather than as a stand-alone epidemiological measure [74]. Gianfredi et al. further showed that non-conventional digital data sources have been used across several communicable tropical and subtropical diseases, including dengue, malaria, WNV, chikungunya, and Zika virus disease [75]. Taken together, this literature supports the interpretation that digital surveillance signals are pathogen- and platform-specific and should be interpreted according to epidemiological context, media visibility, language, and data availability.

In Greece, the WNV-specific pattern is epidemiologically plausible due to its recurrent seasonality and high visibility in open-access data. A critical methodological insight from this study is that digital attention is not a linear measure of total incidence, but a complementary indicator of public information-seeking activity that may increase after periods of heightened epidemiological visibility [51,76]. In the weekly analysis, Greek-language Wikipedia pageviews showed the strongest and most consistent associations with WNND/CNS activity in both 2024 and 2025, particularly when pageviews followed reported WNND/CNS cases by one week. This pattern suggests that Wikipedia activity may reflect sustained information-seeking after increases in epidemiological visibility, official updates, or related media attention. Therefore, Wikipedia pageviews should be interpreted as a Greek-language indicator of public attention and information demand aligned with national surveillance, not as an early-warning or forecasting tool.

Google Trends activity for the term “Ιός Δυτικού Νείλου” also reflected WNV visibility, but the associations were weaker and less consistent than those observed for Wikipedia pageviews. These findings refine, rather than overstate, the role of infoveillance in WNV preparedness. Digital indicators should not be interpreted as direct measures of clinical incidence. Instead, they provide complementary information on when public attention and information demand increase during the transmission season. This distinction is important for risk communication: Google Trends may help identify short-term search interest, whereas Wikipedia pageviews may better capture more sustained educational information-seeking after periods of increased WNND/CNS activity [53,54,55,56,57,77,78,79].

The regional framework in Crete provides field-level preparedness context for interpreting national infodemiological signals. While digital attention at the national level reflects public information-seeking behavior, it does not directly measure vector abundance, environmental risk, or operational readiness. The local One Health framework in Crete, integrating molecular testing, sentinel chicken serosurveillance, entomological surveillance, and citizen-facing reporting, illustrates how digital attention indicators can be interpreted alongside measurable environmental, animal-sentinel, and operational surveillance data [80,81].

At the regional level, EYWA outputs were used operationally as part of the broader preparedness framework in Crete. In 2025, these outputs suggested localized areas of increased vector-related risk, while the MIMESIS model indicated an elevated localized probability of WNV occurrence in Crete. Because no prospectively defined validation was performed, these observations should be interpreted only as supportive operational context and not as formal model validation. More broadly, the regional modeling outputs and the national digital indicators serve different purposes and were not statistically linked in the present study.

The integration of sentinel chicken serosurveillance added a valuable animal-sentinel layer to the West Nile virus (WNV) preparedness strategy. The detection of eight WNV-seropositive sentinel chickens in 2025, following a year of zero seropositive detections, highlights the importance of multimodal surveillance—human, entomological, and animal—even when findings between methods appear to diverge due to sampling timing, spatial coverage, or local ecological heterogeneity. The absence of WNV-positive mosquito pools should not be interpreted as evidence of absent viral circulation, because mosquito-based molecular detection is highly dependent on trapping density, timing, pool composition, local vector infection prevalence, and the micro-focal nature of WNV transmission. Thus, negative mosquito pool testing can coexist with human cases or sentinel-chicken seroconversion, particularly when viral circulation is spatially localized or temporally brief. The documented response to the first locally acquired human WNV case recorded in Crete since 2018 further illustrates how preparedness was translated into field action. Before confirmation of the human case, national WNV activity had already increased clinical and public health vigilance across Greece, while local sentinel chicken serosurveillance in Crete provided animal-sentinel evidence compatible with WNV circulation. These animal-sentinel findings were communicated to the Hellenic National Public Health Organization, which in turn informed health-care services and hospitals, supporting heightened clinical awareness for possible human WNV infection. Following confirmation of the case, regional authorities activated an operational response under the national WNV action framework, including assessment of the likely exposure context, targeted entomological investigation, inspection and control of potential breeding sites, selective vector-control actions where indicated, intersectoral communication, and risk-based area management with relevant public health and blood safety measures. This example shows how national surveillance alerts, animal-sentinel signals, clinical vigilance, epidemiological assessment, and vector-control operations can be linked within a localized WNV preparedness response.

Local vector indicators require cautious interpretation of regional risk. Although the inferential focus of the present study was WNV, the operational pressure from mosquito-borne diseases in Crete is not limited to WNV alone. During the study period, imported malaria and dengue events were recorded locally, and the notification of two additional imported malaria cases by mid-June 2026, although outside the analytical window of this study, further illustrates the continuing relevance of imported vector-borne disease events for island preparedness. These post-study-period observations were not included in the statistical analysis and do not alter the study results; rather, they reinforce the need for sustained clinical vigilance, entomological surveillance, point-of-entry awareness, and risk communication beyond WNV-specific transmission seasons. While adult abundance and larval positivity in 2025 were generally lower than historical averages, such metrics do not preclude localized WNV circulation or focal transmission risk. The regional preparedness approach has also evolved toward year-round operational readiness, including a 12-month cycle for 2026–2028 in line with Ministry of Health guidance [82]. Because mosquito-borne risk extends beyond WNV, the program also includes monitoring of strategic points of entry and oversight of aircraft disinsection certificates for flights arriving from countries or regions with established *Aedes aegypti* populations or other specified risk areas [60,82,83,84,85].

Building upon this operational experience, the regional preparedness framework is being further strengthened in 2026 through a formal Programmatic Agreement with the Laboratory of Clinical Virology at the University of Crete, School of Medicine. This collaboration operates in tandem with the official national surveillance and investigation protocols of the NPHO. By streamlining localized diagnostic confirmation, this arrangement may support earlier activation of public health interventions and blood safety measures, thereby strengthening timely local preparedness and response.

The emergence of tools such as ECDC’s episomer, developed for the early detection of public health threats from social media data, illustrates the growing institutional relevance of digital epidemic intelligence [86,87]. Although episomer was not used in the present analysis, its development supports the broader rationale for integrating digital signals into public health surveillance. Our findings for WNV are consistent with this direction, showing that online information-seeking behavior may complement official surveillance by reflecting public attention during periods of epidemiological activity. Community-based tools, such as the “Kounoopia Watch” initiative under Horizon Europe DxHub, highlight the potential role of citizen observations in complementing official preparedness systems [88]. Such initiatives may complement established surveillance and are conceptually aligned with European data-driven early-warning approaches.

Several limitations should be considered. Google Trends provides normalized rather than absolute search volumes, and the Greek-language WNV series was highly intermittent, with activity concentrated in a small number of non-zero weeks and near-zero values during much of each year. This sparse distribution limits the stability and reproducibility of the Google Trends associations, which should therefore be interpreted with particular caution. Nevertheless, the findings suggest that infodemiological indicators may provide a complementary measure of public information-seeking. Wikipedia pageviews showed a more stable language-specific pattern aligned with national surveillance than Google Trends. However, because reliable per-article reader geolocation is not available through the public Wikimedia Pageviews API, the proportion of pageviews originating outside Greece could neither be quantified nor corrected. Accordingly, the Wikipedia series should be interpreted as Greek-language information-seeking activity aligned with national surveillance, rather than as a geographically restricted measure of online attention among the Greek population. Apart from the exploratory first-difference lag analysis, the study did not include formal time-series modeling of temporal autocorrelation, seasonal adjustment, ARIMA pre-whitening, distributed-lag regression, Granger-causality testing, or adjustment for media coverage. The first-difference analysis showed no leading digital signal: Wikipedia activity remained concurrent with or lagged behind reported cases, whereas the Google Trends correlations were unstable and non-significant. Given the limited number of weekly observations, these results were interpreted descriptively and were not used for formal statistical inference. The observed associations should therefore be regarded as exploratory temporal concordance between surveillance activity and public information-seeking, rather than as evidence of causality, forecasting performance, or independent early-warning capacity. Media coverage and the timing of official surveillance updates were not systematically quantified; consequently, part of the observed digital activity, particularly when Wikipedia pageviews followed WNND/CNS cases, may reflect public responses to increased disease visibility rather than direct responses to epidemiological occurrence. The two-year study window further limits generalizability and did not permit out-of-sample validation across independent transmission seasons. The platform-specific patterns should therefore be considered preliminary evidence from two contrasting WNV seasons in Greece and require confirmation using longer multi-year datasets. Finally, the sentinel chicken component was based on aggregated operational serosurveillance results rather than individual bird-level data; these findings were consequently interpreted as descriptive animal-sentinel preparedness evidence and were not used for spatial or individual-level statistical inference.

## 5. Conclusions

This study identified platform-specific temporal concordance between digital attention and official WNND/CNS surveillance in Greece. In the full-year analyses, Greek-language Wikipedia pageviews showed the strongest associations, particularly when they followed reported cases by one week, whereas Google Trends associations were weaker and less consistent. However, several associations were attenuated in the transmission-period sensitivity analyses: the 2025 Wikipedia associations and the Google Trends associations in both years were not statistically significant. The first-difference lag analysis also identified no leading digital signal. These findings therefore reflect concurrent or lagging public information-seeking and should not be interpreted as evidence of prediction, causality, or independent early-warning capacity.

The findings underscore the pathogen- and platform-specific nature of infodemiology. In the present dataset, WNV was the only mosquito-borne infection with sufficient recurrent seasonality, domestic transmission, weekly surveillance detail, and public health visibility to permit exploratory temporal analysis. Wikipedia provided a more stable language-specific indicator of information-seeking than the highly intermittent Google Trends series. Neither platform can substitute for clinical, laboratory, entomological, or animal-sentinel surveillance.

Analyzed separately from the national digital indicators, the Crete component provides descriptive evidence of regional One Health preparedness. Molecular testing of mosquitoes, sentinel chicken serosurveillance, routine entomological monitoring, citizen-facing reporting, and the documented response to a locally acquired human WNV case illustrate how complementary surveillance information can support field-level vigilance and operational response. This regional evidence does not constitute local validation of the national digital analysis.

Infodemiological monitoring aligned with national surveillance may help identify periods of increased public information demand and inform the timing of risk communication, while regional One Health systems provide the field capacity required for surveillance and targeted response. Future research should use longer multi-year datasets, prospectively specified analytical models, measures of media coverage and official reporting, subnational digital indicators where technically feasible, and out-of-sample validation before assessing any forecasting application. In particular, once longer multi-year series become available, digital attention indicators could be combined with epidemiological, entomological, and environmental data to train machine-learning or early-warning models; however, such applications would require prospective, out-of-sample validation before any operational forecasting use.

## Figures and Tables

**Table 1:** Official national human West Nile virus data in Greece, 2024–2025.

Year	Total Reported WNV Cases	Imported WNV Cases	Locally Acquired WNV Cases	WNND/CNS Cases	Non-CNS Locally Acquired Cases	Deaths	Use in This Study
2024	226	6	220	157	63	35	Main inferential analysis using WNND/CNS cases
2025	98	2	96	76	20	9	Main inferential analysis using WNND/CNS cases

Note: WNND/CNS and non-CNS counts refer to locally acquired cases only (2024: 157 + 63 = 220; 2025: 76 + 20 = 96). Imported WNV cases (6 in 2024, 2 in 2025) were not classified by neuroinvasive status and were excluded from the inferential digital analysis.

**Table 2:** Weekly correlations between WNND/CNS surveillance indicators and digital attention indicators in Greece, 2024–2025.

Year	Digital Indicator	Spearman Lag 0	*p*-Value	Spearman (Digital Precedes Cases by 1 Week)	*p*-Value	Spearman (Digital Follows Cases by 1 Week)	*p*-Value	Pearson Lag 0	*p*-Value
2024	“Ιός Δυτικού Νείλου” Google Trends	0.351	0.010 *	0.348	0.011 *	0.402	0.003 *	0.313	0.022 *
2024	“ιός του Δυτικού Νείλου” Google Trends	0.252	0.069	0.213	0.130	0.213	0.130	0.318	0.020 *
2024	“West Nile virus” Google Trends	NA	NA	NA	NA	NA	NA	NA	NA
2024	“Ιός του Δυτικού Νείλου” Wikipedia	0.750	<0.001 *	0.714	<0.001 *	0.802	<0.001 *	0.816	<0.001 *
2025	“Ιός Δυτικού Νείλου” Google Trends	0.418	0.002 *	0.410	0.003 *	0.452	<0.001 *	0.396	0.003 *
2025	“ιός του Δυτικού Νείλου” Google Trends	0.245	0.077	0.187	0.185	0.297	0.032 *	0.271	0.049
2025	“West Nile virus” Google Trends	0.042	0.767	0.038	0.787	0.055	0.700	−0.068	0.630
2025	“Ιός του Δυτικού Νείλου” Wikipedia	0.718	<0.001 *	0.701	<0.001 *	0.763	<0.001 *	0.630	<0.001 *

Note: *p*-values marked with an asterisk (*) remained statistically significant after adjusting for multiple hypothesis testing using the Benjamini–Hochberg False Discovery Rate (FDR) procedure.

**Table 3:** Summary of local WNV preparedness indicators from Crete, 2024–2025.

Indicator	2024	2025
Major wetland systems monitored	9	13
Settlements covered	747	741
Breeding sites inspected	40,487	40,971
Stormwater drains inspected	>71,420	>70,284
Adult mosquito samplings	576	608
Fixed adult trap sites	32	32
Ad hoc adult trap sites	0	22
Positive WNV mosquito pools	0	0
Sentinel chicken samples	504	511
WNV-seropositive sentinel chickens	0	8
Local human WNV events	0 locally acquired WNV cases	1 locally acquired WNV case
Citizen requests addressed	111	72
Citizen-facing reporting tool	Mosquito Vision^®^	Mosquito Vision^®^
Spatial risk shifts (EYWA/MAMOTH)	Predominantly low-to-moderate risk	Shift to localized high-risk pockets
WNV occurrence output (EYWA/MIMESIS)	No elevated localized probability output reported	Elevated localized probability of WNV occurrence

## Data Availability

Official national surveillance data analyzed in this study are publicly available from the Hellenic National Public Health Organization and the European Centre for Disease Prevention and Control. Google Trends data are publicly available through Google Trends, and Wikipedia pageview data are publicly available through the Wikimedia pageviews platform. Aggregated local preparedness and operational data from Crete are summarized within the article. The full local operational datasets are not publicly available because they derive from regional public health preparedness and mosquito-control records.

## Abbreviations

The following abbreviations are used in this manuscript:

NPHO	Hellenic National Public Health Organization
WNV	West Nile virus
WNND	West Nile neuroinvasive disease
CNS	Central nervous system
ELISA	Enzyme-linked immunosorbent assay
RSV	Relative search volume
REST	Representational state transfer
API	Application programming interface
BAd	Big Data Technologies’ model for Adult mosquitoes
IMBB-FORTH	Institute of Molecular Biology and Biotechnology, Foundation for Research and Technology Hellas
EYWA	Early Warning System for Mosquito-borne diseases
MAMOTH	Earth observation data-driven model for mosquito abundance prediction
MIMESIS	Spatial dynamic model for West Nile virus
ARIMA	Autoregressive integrated moving average
NOA	National Observatory of Athens
Ecodev	Ecodevelopment S.A.
FDR	Benjamini–Hochberg False Discovery Rate
